# Rethinking palliative care in a public health context: addressing the needs of persons with non-communicable chronic diseases

**DOI:** 10.1017/S1463423620000328

**Published:** 2020-09-15

**Authors:** Chariklia Tziraki, Corrina Grimes, Filipa Ventura, Rónán O’Caoimh, Silvina Santana, Veronica Zavagli, Silvia Varani, Donatella Tramontano, João Apóstolo, Bart Geurden, Vincenzo De Luca, Giovanni Tramontano, Maria Rosaria Romano, Marilena Anastasaki, Christos Lionis, Rafael Rodríguez-Acuña, Manuel Luis Capelas, Tânia dos Santos Afonso, David William Molloy, Giuseppe Liotta, Guido Iaccarino, Maria Triassi, Patrik Eklund, Regina Roller-Wirnsberger, Maddalena Illario

**Affiliations:** 1 Israel Gerontological Data Center, Hebrew University of Jerusalem, Jerusalem, Israel; 2 MELABEV – Community Clubs for Elders, Jerusalem, Israel; 3 Public Health Agency of Northern Ireland, Belfast, UK; 4 The Health Sciences Research Unit: Nursing, Nursing School of Coimbra, Coimbra, Portugal; 5 Department of Medicine, Clinical Sciences Institute, National University of Ireland, Galway, Ireland; 6 Department of Economics, Management, Industrial Engineering and Tourism, Institute of Electronics and Informatics Engineering of Aveiro, University of Aveiro, Aveiro, Portugal; 7 Psycho-Oncology Unit, ANT Italia Foundation, Bologna, Italy; 8 ANT Italia Foundation, Bologna, Italy; 9 Department of Molecular Medicine and Medical Biotechnology, Federico II University of Naples, Naples, Italy; 10 Department of Nursing, Nursing School of Coimbra, Coimbra, Portugal; 11 Nursing and Midwifery, Faculty of Medicine and Health Sciences, University of Antwerp, Antwerp, Belgium; 12 Research and Development Unit, Federico II University Hospital, Naples, Italy; 13 Hospital Care Division, General Directorate for Health, Campania Region, Naples, Italy; 14 Clinic of Social and Family Medicine, School of Medicine, University of Crete, Heraklion, Crete, Greece; 15 Department of Social Medicine, School of Medicine, University of Crete, Heraklion, Crete, Greece; 16 Andalusian Public Foundation Progress and Health (FPS), Seville, Spain; 17 Interdisciplinary Health Research Center (CIIS), Institute of Health Sciences, Portuguese Catholic University, Lisbon, Portugal; 18 Faculty of Pharmacy, Center for Pharmaceutical Studies, University of Coimbra, Coimbra, Portugal; 19 Centre for Gerontology and Rehabilitation, School of Medicine, University College of Cork, Cork, Ireland; 20 Department of Biomedicine and Prevention, University of Rome Tor Vergata, Rome, Italy; 21 Department of Advanced Biomedical Sciences, Federico II University of Naples, Naples, Italy; 22 Department of Public Health, Federico II University of Naples, Naples, Italy; 23 Department of Computing Science, Umeå University, Umeå, Sweden; 24 Department of Internal Medicine, Medical University of Graz, Graz, Austria; 25 Department of Public Health, Federico II University of Naples, Naples, Italy; 26 Health Innovation Division, General Directorate for Health, Campania Region, Naples, Italy

**Keywords:** integrated, multimorbidity, non-communicable chronic diseases (NCCDs), palliative care, public health

## Abstract

Non-communicable chronic diseases (NCCDs) are the main cause of morbidity and mortality globally. Demographic aging has resulted in older populations with more complex healthcare needs. This necessitates a multilevel rethinking of healthcare policies, health education and community support systems with digitalization of technologies playing a central role. The European Innovation Partnership on Active and Healthy Aging (A3) working group focuses on well-being for older adults, with an emphasis on quality of life and healthy aging. A subgroup of A3, including multidisciplinary stakeholders in health care across Europe, focuses on the palliative care (PC) model as a paradigm to be modified to meet the needs of older persons with NCCDs. This development paper delineates the key parameters we identified as critical in creating a public health model of PC directed to the needs of persons with NCCDs. This paradigm shift should affect horizontal components of public health models. Furthermore, our model includes vertical components often neglected, such as nutrition, resilience, well-being and leisure activities. The main enablers identified are information and communication technologies, education and training programs, communities of compassion, twinning activities, promoting research and increasing awareness amongst policymakers. We also identified key ‘bottlenecks’: inequity of access, insufficient research, inadequate development of advance care planning and a lack of co-creation of relevant technologies and shared decision-making. Rethinking PC within a public health context must focus on developing policies, training and technologies to enhance person-centered quality life for those with NCCD, while ensuring that they and those important to them experience death with dignity.

## Introduction

Non-communicable chronic diseases (NCCDs), such as diabetes, cancer, dementia and heart disease, rank second on the World Health Organization (WHO) list of global health priorities (World Health Organization, 2019a). Persons with NCCDs account for ≥70% of all deaths worldwide (Centeno and Arias-Casais, 2019; World Health Organization, 2019b). Palliative care (PC) is essential to quality living as well as to quality death for persons with NCCDs, and the concepts and services related to its provision are part of the integrative care approach to health care that includes health promotion even in the presence of established disease (Mittelmark *et al.*, 2017). The WHO definition of PC puts emphasis on enhancing quality of life (QOL) for patients and their families through the prevention/relief of suffering by means of early identification, comprehensive assessment and treatment of pain and other physical, psychosocial and spiritual suffering. Achieving these goals requires an integrative approach. This includes the promotion of appropriate policies, adequate access to treatment and interventions such as drug availability, education of both healthcare workers and the public, and the implementation of generalist PC services at all levels of society (Abel *et al.*, 2018). While not denying the reality of death, PC offers a positive approach for living life to the full, even for those with NCCDs (Tiberini and Richardson, 2015; World Health Organization, 2019b). The number of persons potentially benefitting from integrated PC in chronic care management is estimated to rise from 25 to 47% in 2040 (Centeno *et al.*, 2018).

The 2016 review of public health approaches to PC by Colleen Dempers and Merryn Gott recognized three main paradigms in PC and public health, disciplines with overlapping boundaries: health promotion approaches, WHO approach and population-based approaches (Dempers and Gott, 2017).

The aim of this paper is to stimulate a discussion on how to promote a paradigm shift towards the deployment of a public health approach to delivering PC for persons with NCCD that addresses the spectrum from ‘end of life’ to ‘quality of life while living longer’ (Evans *et al.*, 2019). We will present a new concept of integration of PC through horizontal and vertical actions, applicable within any health and social care system, with a person-centered PC approach for people with NCCDs, their families and caregivers and supporting not only a quality of death but also that of life with persons with NCCDs.

Our aim is to build on the work presented in the European Association for Palliative Care Atlas (Arias-Casais *et al.*, 2019) and the current document on PC by the WHO (World Health Organization, 2019b) via collaboration in the A3 action group on ‘Lifespan Health Promotion & Prevention of Age Related Frailty and Disease’ of the European Innovation Partnership on Active and Healthy Ageing (EIP/AHA). We will discuss (primarily within an EU context) the enablers, barriers and gaps in shifting PC toward a person-centered public health approach that emphasizes the early detection of PC needs to maintain QOL (Scheerens *et al.*, 2020) and allows the integration of PC into chronic care management. We anticipate that this discourse will stimulate readers to consider new policy and pathways for integrating chronic care management into macro-, meso- and micro-levels of care systems.

## Factors important to build a successful public health model of PC in the context of people with NCCD and their management

In 2016, DeSalvo *et al.* (2016) sounded a call to action for public health to ‘boldly expand the scope and reach of public health to address all factors that promote health and well-being, including those related to economic development, education, transportation, food, environment, and housing’. This extended scope of public health is important in the development of a successful public health model for PC.

### Horizontal factors

**Figure 1.**
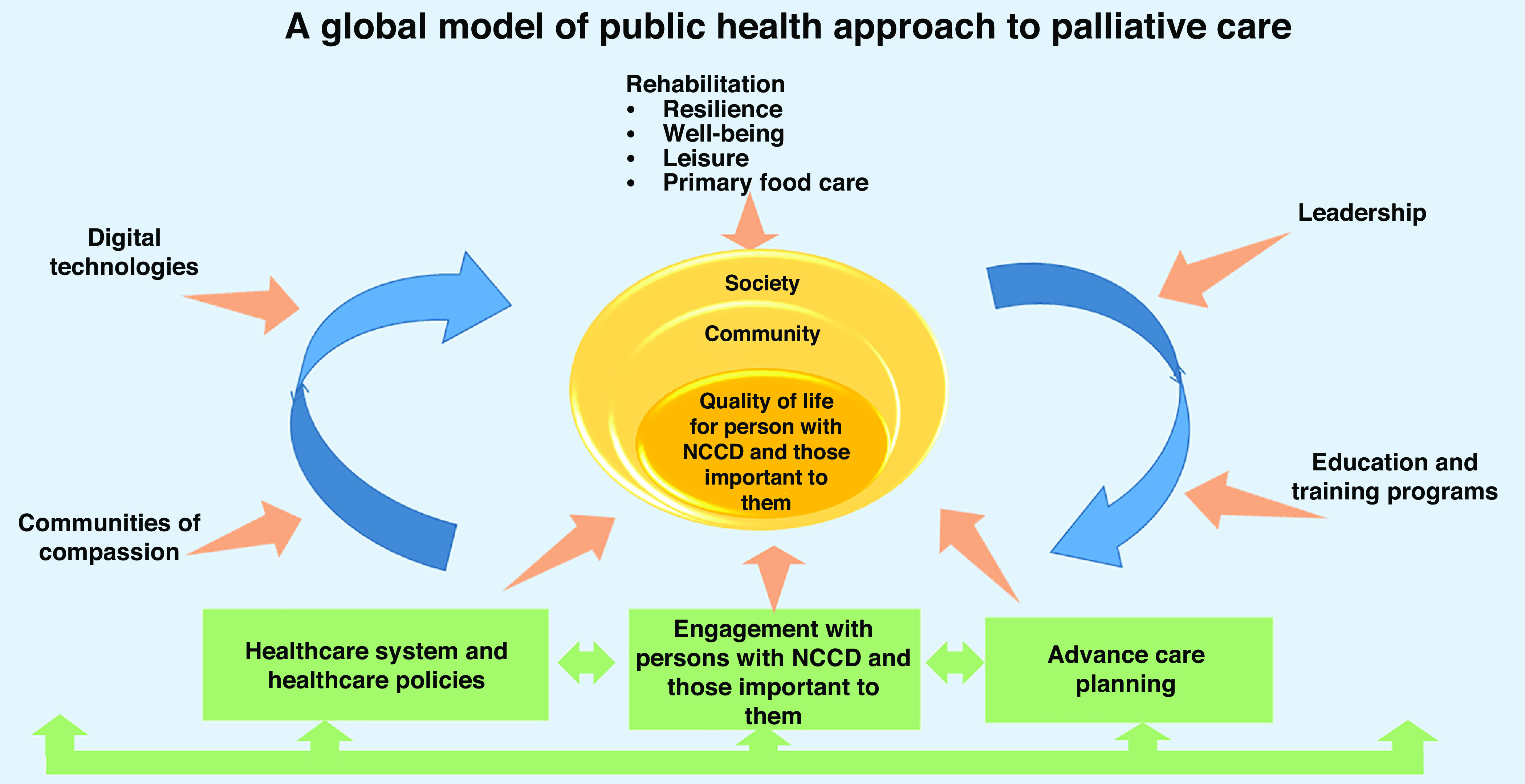
Rethinking palliative care for chronic diseases

As previous literature has pointed out (Abel *et al.*, 2018), clinical factors alone are not sufficient to address the social aspects of PC. We aim to create a framework for improving PC for persons with NCCDs (Pfaff *et al.*, 2020), through person-centered care model. Figure 1 depicts the domains covered in this paper.

We review factors that operate on the horizontal level, that is, process-related factors, the primary pathways through which persons with CD usually pass, and discuss factors through a vertical lens that focuses primarily on knowledge and information in their broadest sense (Heeringa *et al.*, 2020).

Since the publication of the WHO public health model, a guideline for governments to help implement and integrate PC into healthcare systems, there has been a remarkable expansion in access to PC across European countries (Callaway *et al*.*et al*., 2018). Implementation has mainly focused on institutional care, but it is also offered in primary care models in different European member states. PC can take advantage of ‘lessons learnt’ from these models and integrate these concepts on a broader, community level, while maintaining a person-centered approach. The following section highlights factors supporting successful integration of PC into a chronic care model at the macro-level.

#### Healthcare systems and health policies

In the majority of countries, PC is only provided in the last month of life, rather than in the context of living with NCCD (Arias-Casais *et al.*, 2019).

The WHO public health model promotes the implementation of PC approach at multiple levels of society. This requires the successful coordination of local, regional and national organizations and communities to identify PC needs and promotion of a palliative rehabilitation approach for persons with NCCDs. This can maximize their physical and mental well-being, promote resilience and maintain QOL (Greenlund *et al.*, 2012). In 2000, the National Council for Hospice and Specialist Palliative Care Services outlined the aims of palliative rehabilitation as to ‘maximize patients’ ability to function, to promote their independence and to help them adapt to their condition’.

Extension of PC beyond healthcare systems via a ‘compassionate communities’ approach will shift the focus from end-of-life care towards ‘early’ PC and health promotion despite disease conditions (World Health Organization, 2014; Abel *et al.*, 2018; Whitelaw and Clark, 2019).

In 2018, the Parliamentary Assembly of the Council of Europe published a resolution calling on Member States to take measures to strengthen PC services, thereby ensuring access and provision to quality PC for both adults and children (Parliamentary Assembly of the Council of Europe, 2018; Carlson *et al.*, 2015).

PC networks are also recognized as important structures to support the integration of care by bringing together key professionals to meet the complex needs of those requiring PC (Bainbridge *et al.*, 2010). These networks are ideally suited for those with NCCD given their complex care needs. It is also likely that such networks can facilitate an overarching public health approach to PC and improve integration of care in a cost-effective and efficacious way (Bainbridge *et al.*, 2010).

#### Advance care planning

Advance care planning (ACP) describes the formalized process of meeting, discussing and documenting individuals’ preferences for their future care needs for a time when they are no longer able to participate meaningfully in decision-making (Crowther and Costello, 2017). Being able to express one’s future preference for treatment and engage in shared care planning is an integral part of PC.

ACP should involve multiple stakeholders including, but not limited to, the individuals themselves, those important to them and interdisciplinary healthcare and social care professionals. The focus is on the co-production as a means to achieve inclusive, holistic and person-centered ACP.

It is recommended that ACP begin at an early stage of chronic disease. Little research, however, is available on how to achieve this objective. This is reflected by generally low levels of uptake in all parts of the world (Mullick *et al.*, 2013; Osborn *et al.*, 2014) that may be related to cultural taboos and beliefs (Con, 2007; Rao *et al.*, 2014). It is important to assess public health aspects of the process that may support and encourage stakeholders to open a dynamic dialogue at the earliest appropriate stage. This has been described as ‘up-streaming and normalizing’ ACP discussions (Prince-Paul and DiFranco, 2017).

#### Patients and their families

The WHO recommends approaching persons with NCCD and their caregivers as a ‘unit of care’, focusing on the well-being of the patient–caregiver dyad rather than just on the patient. It is important to recognize caregivers’ needs, and supporting family caregivers has pre- and post-bereavement benefits (Aoun *et al.*, 2017). A ‘family caregiver’ in this context is anyone providing any type of physical and emotional care for an individual at home. While family caregivers can be considered an extension of the healthcare team, they often do not feel adequately prepared for their role, which may impact on their physical and mental well-being (Robison *et al.*, 2009; Rohleder *et al.*, 2009) and even cause them to become the next cohort of persons with NCCDs. PC research, policy and practice must invest in the needs of these caregivers, because supporting caregivers is a cost-effective long-term care investment: caregivers reduce demand for institutionalization and reliance on public health programs (World Health Organization, 2014). Even when family members do not take on caregiver roles, their needs must be addressed (Mehta *et al.*, 2009), as the illness of one member impacts on all other members.

Social support is a protective factor in grief (Burke and Neimeyer, 2013), and social relations have ‘a larger impact on reducing mortality than any other existing intervention’ (Holt-Lunstad *et al.*, 2010). Compassionate communities and community caring networks (Aoun *et al.*, 2018) have been developed in different countries (McLoughlin *et al.*, 2015), where family members have opportunities to share their grief experiences outside family so as to avoid overloading each other (Hooghe *et al.*, 2011).

PC guidelines propose that bereavement support be offered according to need (World Health Organization, 2017). However, in practice, bereavement is often not recognised as being an integral part of PC, and there tends not to be formal guidelines/standards nor the use assessment tools to support its management and identification (Guldin *et al.*, 2015). Future guidelines should include targeted support, based on personalized needs, risk assessment measures and clear evidence of efficacy (Breen *et al.*, 2014).

### Vertical factors

#### Primary food care

Primary food care (PFC) is all the care that is spent on the balanced composition, appropriate preparation and daily providing of regular meals. Optimal PFC is the result of integrated care in which many stakeholders are involved, including the persons themselves and their family caregivers. PFC was first described by EIP/AHA (Illario *et al.*, 2016) as an indispensable part of the food-and-nutritional approach to nutritional frailty in elderly (Illario *et al.*, 2016). However, all aspects of PFC are also equally as important in PC: QOL, symptom management, patient autonomy and attention to psychosocial, emotional and spiritual aspects, including cultural needs, not only for the persons with PC needs but also those important to them.

As the person’s NCCD progresses, healthy eating guidelines recommended for the general population are no longer appropriate due to the effects of illness and treatment on the body and potential disturbances in appropriate food intake (Klein *et al.*, 2011; Pilgrim *et al.*, 2015). The aim of PFC in this stage of PC is to minimize food-related discomfort and maximize food enjoyment with respect to the individuals’ caloric needs, and it should be accompanied by early identification of any food-related problem (Hutton *et al.*, 2007; Boltong *et al.*, 2014). The employment of PFC interventions needs to be discussed with the person and her family with respect to cultural and religious sensitivities (Maher and Hemming, 2005).

In the late stage of PC, the focus of PFC interventions progressively shifts to maximizing food enjoyment, with less emphasis on caloric and nutrient needs. A specific food or a distinct taste might even evoke reminiscence (Nyatanga, 2015), memories of happy moments from the past. Food has a much greater significance than merely provision of nutrients, not only for the person with the PC need but also those important to him.

PFC innovations, such as personalizing meals based on preference and symptom management, are not yet common practice in health care and social care (Holder, 2003; Amano *et al.*, 2018), and thus an area which has been minimally addressed in scientific literature.

#### Resilience, well-being and leisure

Between physical health and subjective well-being exists a two-way relation: poor health leads to a reduced subjective well-being, while high well-being can reduce physical health impairments and is associated with longer survival (Veenhoven, 2008; Ryff, 2014; Steptoe *et al.*, 2015; Diener *et al.*, 2015, 2017; Martín-María *et al.*, 2017).

Resilience is the capacity of coping with changes and bouncing back from stress or adversity. It is associated with longevity, lower rates of depression and greater satisfaction with life and helps to maintain and promote mental health (Earvolino-Ramirez, 2007; Fletcher and Sarkar, 2013; McEwen, 2016).

Social isolation and loneliness are risk factors for NCCDs (Malcolm *et al.*, 2019), but they can also be their consequence, worsening the disease and limiting the success of therapeutic interventions. Fighting social isolation and loneliness is a key factor in limiting the negative outcomes of NCCDs. Leisure activities may provide for persons with NCCDs a way to better cope with their disease and social isolation (Rapacciuolo *et al.*, 2016; Denovan and Macaskill, 2017; Goulding, 2018) and motivate them to address other aspects of their activities of daily living (Cuypers *et al.*, 2012; Hansen *et al.*, 2015; Paggi *et al.*, 2016). Leisure activities should have a key role in PC strategies for NCCDs through a palliative rehabilitation model (Coelho *et al.*, 2017; Zeilig *et al.*, 2018).

## Enablers to enable a public health approach

### Digital technologies to enable a public health approach

Innovative digital solutions for public health approach to PC facilitate sharing of information relevant to the health of the person, improve the collective impact of the support provided and relieve caregiver strain (Mayahara *et al.*, 2017; Sirintrapun and Lopez, 2018). However, there is little evidence as yet of the impact of technology on the QOL in NCCD (Arris *et al.*, 2015), and evaluation or implementation studies are still very scarce (Curtis *et al.*, 2018; Phongtankuel *et al.*, 2018).

The development, deployment and evaluation of information and communication technology (ICT) and information systems in PC call for a patient- and family-centered multi-stakeholder approach – including the patient and people important for him/her, formal and informal caregivers, policy makers and payers with their needs, expectations and limitations – which is often absent from planning and daily practice.

Technologies have been clustered according to their contribution to four pressing needs and aspirations of persons with NCCD at the end of life and the people important for them: feeling in control of their lives, telling their story, staying connected and communicating with caregivers (Portz *et al.*, 2018).

Although potentially advantageous and feasible, video consultations lack evidence on general PC, non-cancer cases and countries in a low-socioeconomic status (Jess *et al.*, 2019).

A variety of new media, including voice-assisted technology, digital diaries and virtual reality, can enable older adults to record and share their personal narratives, knowing their life story survives them.

Artificial agents, such as robots, embodied conversational agents and chatbots, have been shown to reduce NCCD loneliness by providing social support (Loveys *et al.*, 2019). A number of digital solutions are available to help coordinate and connect a network of caregivers and services.

Technology can also facilitate the widespread dissemination of core knowledge and skills that many generalist clinicians/professionals critically need, including communication skills, via e-learning initiatives (Bates, 2016).

There exist a number of examples of how digital solutions have been deployed. Digital storytelling with VoiceThread technology has been used to promote deeper understanding in nursing students about PC concepts (Price *et al.*, 2015).

QDACT, a technology-based quality monitoring system for PC, combines patient-reported data with critical PC steps (ACP, inclusion of caregivers) in order to document care quality, link measures of QOL to outcomes in PC and help health professionals to uncover and address unmet needs (Bates, 2016).

Health technology allows a centralized Hub where easy-to-read educational materials and treatment guidelines for persons in hospice-based care and their caregivers are readily accessible by a smartphone or a tablet (Phongtankuel *et al.*, 2018).

Globally, there are a number of website resources for healthcare providers and the public. E Hospice (https://ehospice.com/), based in the UK, is a news and information resource that brings global news, commentary and analysis concerning hospice, palliative and end-of-life care, offering a single point of access to good practice from around the world. The All Ireland Institute of Hospice and Palliative Care (https://aiihpc.org/our_work/the-palliative-hub/) and The Canadian Virtual Hospice (http://www.virtualhospice.ca/) join several actors of PC context in a common space for information use (Phongtankuel *et al.*, 2018).

Integrating technological advances into a specialty such as PC is not without its challenges: interaction with impersonal technology; designing adequate, intuitive and user-friendly technology and storing and protection of confidential information (Phongtankuel *et al.*, 2018; Jess *et al.*, 2019). Reimbursement is also a challenge, as under many funding schemes only face-to-face encounters are counted, not screen time.

### Transforming PC through leadership, awareness, education and training programs

#### Professional staff

Professional, individual and community education and training are important action areas for integrating and promoting the core components of global PC initiatives and programs. Project ECHO (https://echo.unm.edu/) is such a leading action comprising a lifelong learning and guided practice model focused on improving medical education and increasing workforce capacity and self-efficacy to provide best-practice care and reduce health disparities, through multidisciplinary knowledge-sharing, networking and technology utilization (Ballesteros *et al.*, 2014; Carrasco *et al.*, 2015).

Availability of adequate tools and training can have a substantial impact on professional sensitization towards PC. In a randomized controlled trial, trained general practitioners demonstrated significant increases in the rates of identification and provision of multidimensional care compared to controls (Thoonsen *et al.*, 2019). Additionally, early training on PC seems to have positive effects on future professionals’ competences (Parikh *et al.*, 2017; El-Sourady *et al.*, 2019). These examples highlight the core role of all-level education and training in PC transformation (Rotar Pavlič *et al.*, 2019).

Specially needed are synergies among curricula to develop a public health model of PC in low resource setting with a growing burden of NCCDs (Lionis *et al.*, 2011).

#### Creating communities of compassion: public

This section includes two key concepts that are still challenging, health and social care services and policies. The first concerns the term ‘compassion’, a universally accepted definition still under debate. The second refers to communities playing a core role in the success of primary care (World Health Organization, 2018).

The emerging concept of compassionate care is broad and extends to palliative and end-of-life care. Based on Kellehear’s definition (Kellehear, 1999), compassionate care recognizes that ‘*all-natural cycles of sickness and health, birth and death, and love and loss occur every day within the orbits of its institutions and regular activities*’. Despite the availability of several other formulations of this definition, all agree that ‘*it is everyone’s responsibility to care for each other during times of crisis and loss, and not simply the task of health professionals*’ (Tompkins, 2018).

The importance of creating communities attracts a worldwide interest, also in the context of the contribution of public health to PC. An implementation guide for community approaches to end-of-life care has been developed (Compassionate Communities, 2018), according to which ‘*compassionate communities could care for people in all phases of end of live, respect and response to the needs and wishes of the dying person and their families, promote leadership embedded within the local community, work alongside service providers to support people at end of life and their families and careers*’. It also illustrates how compassionate communities could create community partnerships, raise awareness, activate community groups and provide advocacy and policy.

In settings where primary care is still in development and the notion of compassionate care has not been incorporated, there is a need for preparatory work. A key step would be to explore the role of Health Sciences schools and institutions in mobilizing people and communities to understand palliative and end-of-life needs. Introducing courses on clinician–patient relationship and compassionate care with the involvement of patients’ organizations has already been proven efficient (Lionis *et al.*, 2011). Investing in training of future health and social care professionals (Frilund *et al.*, 2013) within the community setting and promoting of collaboration with community groups and stakeholders are also important steps.

### Reference regions and twinning

Ensuring adequate support and services to meet the growing demands of an aging population requires a paradigm shift away from reactive to proactive disease management (Porter and Teisberg, 2006; Busse and Blümel, 2010; Ellis *et al.*, 2011). Implementing strong integration between levels of care and between communities, social sector and health sector is pivotal to provide appropriate services that take advantage of interdisciplinary competences and skills.

Increasing evidence is now available for the effectiveness of digital solutions in supporting integrated care, improving health outcomes and changing management of social and health systems, while increasing accessibility and sustainability (Lionis *et al.*, 2016; Menon *et al.*, 2019; Mitchell and Kan, 2019; Tai-Seale *et al.*, 2019; Turkki *et al.*, 2019). Most importantly, to create this integration, consumers and those with NCCD need to be involved in the technological developments and policy generation. This will empower people to take charge of their health and well-being according to their holistic needs.

Nonetheless, such examples are still fragmented throughout the EU. To speed up large-scale adoption, inter-regional collaborations, such as the ScaleAHA study (http://www.scale-aha.eu/home.html) supported by the European Commission are pivotal, as they allow peer-to-peer adaptations to local socio-cultural, organizational and economic contexts.

Within the ScaleAHA initiative, the ‘2016 Transfer of Innovation Scheme’ was carried out, supporting 20 pairs of regions (26 RSs from 13 European countries) to learn from one another and scale up digitally enabled innovative ICT solutions in active and healthy aging. Inter-regional collaborative activities have also been fostering the use of European Structural and Investment Funds to secure the necessary funding to scale up.

### The importance of leadership

Another, often overlooked but equally important, enabler of developing a sustainable public health approach to PC is strong leadership and management (Fraser *et al.*, 2017). Collective leadership working across sectors and with all key stakeholders is required, with a move away from the traditional siloed approach to providing care management (Fraser *et al.*, 2017). Good governance in this setting should include oversight of networks, engagement with stakeholders, management of resources and the instigation of continuous improvement initiatives (Carlson *et al.*, 2015).

## A new model for a public health approach to PC for NCCDs

So, what could this new vision of a strong and integrated public health approach to PC for those living with NCCDs look like? We have outlined some of the important drivers and enablers for the shift away from a health and social care-based system to a more integrated healthcare pathway adopting an overarching public health that includes all key stakeholders (primary care health professionals, community-based organizations and networks of persons with NCCDs, family, formal and informal caregivers, etc.).

The primary care team is key to the concept of creating an integrative public health model. Figure 1 shows the concept based on recent evidence from the literature, good practice models from the EIP/AHA as well as consensus among experts involved in the work presented in this publication.

Based upon the integrated care concept of Kaiser Permanente (Arias-Casais *et al.*, 2019), the majority of action is located in primary care. Palliative home care can be considered an integrated system of social and health services provided as a continuum, allowing persons with NCCDs to stay in their own life environment as long as possible. When home care services are activated upon hospital discharge with concomitant monitoring (protected discharge), they allow to contain the number and the duration of hospital stays, at the same time ensuring the best assistance possible. ‘Beyond Silos’ is an EU project that addresses the issue of de-hospitalization (Pascale *et al.*, 2016; Visco *et al.*, 2017, 2018).

Integrated care services, closely oriented to the needs of patients/users, interdisciplinary and anchored in community and home care settings can benefit informal caregivers (Tziraki-Segal *et al.*, 2019, Pfaff *et al.*, 2020). In such environments, ICT generally facilitates transfer of information, eliminating redundant paperwork and monitoring.

During implementation of integrated PC in communities, it is recommended to evaluate sustainability of the efforts undertaken and those achieved through the discussion and adaptation of process indicators, especially in the first cycle of implementation. One of the major challenges lying ahead of us is to assess how evidence-based medicine can be applied in public health for the digital transformation of health and care (Grilli *et al.*, 2000; Burgers *et al.*, 2003; Brownson *et al.*, 2009). To this purpose, we should orient our efforts towards the integration of the best available evidence with the knowledge and inputs from stakeholders and experts to identify how to best address people’s health needs within NCCDs. Data from observational studies, surveillance and modeling will provide the evidence base to further progress the implementation of innovative approaches into public health. A variety of methods for reporting, assessing and grading evidence are available (Weightman *et al.*, 2005; Boyd and Bero, 2006; Moynihan *et al.*, 2008).

The collection of data is pivotal to allow their critical evaluation to support sound decision-making, and this not only concerning the effectiveness of an intervention but also other domains such as privacy, safety, cost, economic evaluation, ethical issues, as well as organizational, social and legal aspects. These domains are not traditionally included in most medical research, but in the case of NCCDs they are crucial for making the shift to public health model of PC, to reflect the holistic, whole system approach and to inform optimal models which could be developed within the framework of sustainable economic policies (Bainbridge *et al.*, 2010).

To ensure quality of care and effectiveness of service, there needs to be the development of global health policies; the creation of teams with the skills to collate and utilize health data and develop population needs assessment; the provision of the appropriate and timely intervention including access to medication; and educational efforts aimed at patients, healthcare and social professionals and policy makers, especially at the regional level where the cultural and spiritual dimensions can be more readily addressed (Capelas *et al.*, 2017a, 2017b; Gómez-Batiste *et al.*, 2017a, 2017b).

## Conclusions

Integration of PC for the management of people with NCCDs at a public health level requires engagement with all stakeholders from policy to practice, including those with NCCDs and those caring for them.

Whilst there will be a requirement to have a population approach at policy level, the identification of individual needs, expectations and wishes in order to address them coherently, with available assets and resources, is key throughout all levels of care in healthcare systems.

Building on the concept of ACP, provision of tailored primary food supply and strengthening of formal and informal caregivers, authors first present a new concept of integrated PC to be applied for those presenting with NCCDs.

The engagement with not-for-profit organizations and charities is pivotal for sharing a vision and starting the discussion on the development of a regional compassionate care plan. Collaborative approaches to scale up the adoption of innovations in health and care service provision, such as compassionate communities which are developing at local/regional, national and international levels, are key to ensure that change management is ignited at policy makers level and transferred downstream for implementation.

Peer-to-peer interactions and positive engagement with all stakeholders, with a common effort of implementing transformation in the delivery and provision for care, support and services along the entire chain of collaborations, are required, as opposed to exclusion or competitiveness, which can delay and stifle progress and compromise the quality of care provided to those with NCCDs and their caregivers. This also facilitates experts to overcome boundaries between authorities and through an interdisciplinary approach narrow gaps in quality care that often underpin silos between organizations and sectors.

We recommend a global model of public health approach to PC which also incorporates the long-term caregiving role and manages the factors that influence the informal caregivers’ ability to cope with their role by designing and implementing early supportive interventions, such as education and community support (Janse *et al.*, 2014).

We conclude that the aim of rethinking PC in a public health context will help improve and promote QOL for people and those important to them, while living with NCCD. Thus, the focus should shift from preparing for a good death to living well until you die.
