# Rethinking palliative care in a public health context: addressing the needs of persons with non-communicable chronic diseases – CORRIGENDUM

**DOI:** 10.1017/S1463423620000602

**Published:** 2020-12-10

**Authors:** Chariklia Tziraki, Corrina Grimes, Filipa Ventura, Ronan O’Caoimh, Silvina Santana, Veronica Zavagli, Silvia Varani, Donatella Tramontano, João Apóstolo, Bart Geurden, Vincenzo De Luca, Giovanni Tramontano, Maria Rosaria Romano, Marilena Anastasaki, Christos Lionis, Rafael Rodríguez-Acuña, Manuel Luis Capelas, Tânia dos Santos Afonso, D. William Molloy, Giuseppe Liotta, Guido Iaccarino, Maria Triassi, Patrik Eklund, Regina Roller-Wirnsberger, Maddalena Illario

In the original article, the author affiliation for Tânia dos Santos Afonso was incorrectly listed. The correct affiliation should be as follows:

Interdisciplinary Health Research Center (CIIS), Institute of Health Sciences, Portuguese Catholic University, Lisbon, Portugal

The Authors would like to apologies for this mistake. The affiliation has been updated in the originally published article.
